# The neurochemical basis of human cortical auditory processing: combining proton magnetic resonance spectroscopy and magnetoencephalography

**DOI:** 10.1186/1741-7007-4-25

**Published:** 2006-08-03

**Authors:** Peter Sörös, Nikolaus Michael, Melanie Tollkötter, Bettina Pfleiderer

**Affiliations:** 1 Department of Imaging Research, Sunnybrook Health Sciences Centre, Toronto, ON, Canada; 2 Department of Psychiatry, University Hospital Münster, Münster, Germany; 3 Department of Clinical Radiology, University Hospital Münster, Münster, Germany

## Abstract

**Background:**

A combination of magnetoencephalography and proton magnetic resonance spectroscopy was used to correlate the electrophysiology of rapid auditory processing and the neurochemistry of the auditory cortex in 15 healthy adults. To assess rapid auditory processing in the left auditory cortex, the amplitude and decrement of the N1m peak, the major component of the late auditory evoked response, were measured during rapidly successive presentation of acoustic stimuli. We tested the hypothesis that: (i) the amplitude of the N1m response and (ii) its decrement during rapid stimulation are associated with the cortical neurochemistry as determined by proton magnetic resonance spectroscopy.

**Results:**

Our results demonstrated a significant association between the concentrations of N-acetylaspartate, a marker of neuronal integrity, and the amplitudes of individual N1m responses. In addition, the concentrations of choline-containing compounds, representing the functional integrity of membranes, were significantly associated with N1m amplitudes. No significant association was found between the concentrations of the glutamate/glutamine pool and the amplitudes of the first N1m. No significant associations were seen between the decrement of the N1m (the relative amplitude of the second N1m peak) and the concentrations of N-acetylaspartate, choline-containing compounds, or the glutamate/glutamine pool. However, there was a trend for higher glutamate/glutamine concentrations in individuals with higher relative N1m amplitude.

**Conclusion:**

These results suggest that neuronal and membrane functions are important for rapid auditory processing. This investigation provides a first link between the electrophysiology, as recorded by magnetoencephalography, and the neurochemistry, as assessed by proton magnetic resonance spectroscopy, of the auditory cortex.

## Background

Hearing is one of the fundamental and most important abilities of the vertebrate nervous system. The neurophysiology of the auditory system is increasingly well understood, however, little is known about the associations between the electrophysiology and the neurochemistry of the auditory cortex. One way of assessing auditory function is by measuring the amplitude and the decrement of the N1m wave, the major peak of the late auditory response, during rapidly successive presentation of acoustic stimuli. Recurrent acoustic stimulation is associated with characteristic short- and long-term decreases in the response of auditory neurons as seen by electrophysiologic recordings [1-3] and by functional magnetic resonance imaging [4,5]. The decrement of auditory evoked responses with rapid stimulation, also known as habituation or sensory gating, is believed to represent cortical filtering of irrelevant input [6].

Recent studies have investigated the effects of various psychopharmacological agents on cortical auditory processing [7]. A single dose of haloperidol, a dopamin D2-receptor antagonist, affected the mismatch negativity, an event-related potential to deviating acoustic stimuli, but had no effect on the amplitude and latency of the N1, the electric counterpart of the N1m [8]. Administration of scopolamine, a centrally acting cholinergic antagonist, increased the latency of the N1m peak in young [9] and old [10] individuals. Alcohol consumption similarly increased the N1m latency and decreased the N1m amplitude [11]. Infusion of ketamine, an N-methyl-D-aspartate (NMDA) antagonist, resulted in significant increase of the N1 peak amplitude without affecting the N1 latency [12]. In addition, serotonergic neurotransmission plays an important role in the regulation of auditory processing [13].

In the present study, magnetoencephalography (MEG) and proton magnetic resonance spectroscopy (^1^H-MRS) were performed to assess the relationship between the electrophysiology and the neurochemistry of the left auditory cortex. As MEG allows the recording of cortical magnetic fields with supreme temporal resolution [14], this technique is ideally suited for the investigation of the exact timing of neural functions [15]. The recording of auditory evoked fields (AEF) has a high reliability (test-retest reproducibility) [14] and a high validity (consistency with intracranial recordings) [16]. ^1^H-MRS provides an opportunity to assess the regional neurochemistry of the brain *in vivo *[17]. We tested the hypotheses that: (i) the amplitude of the N1m wave, the major component of the long-latency auditory evoked responses and (ii) the decrement of this response during rapid stimulation are associated with brain metabolites measurable by ^1^H-MRS.

## Results

### Magnetoencephalography

**Figure 1:**
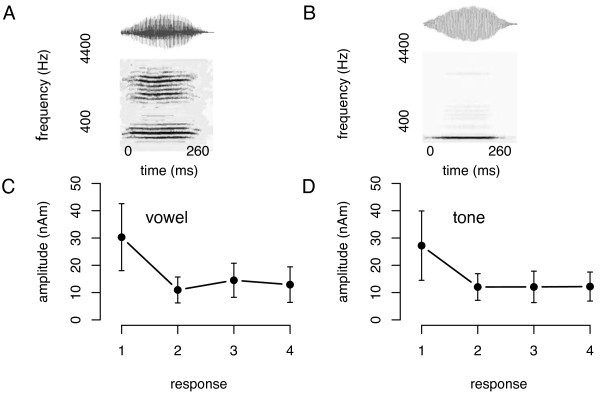
**N1m as a function of stimulus type**. (**A, B**) Acoustic waveforms (upper graphs) and spectrograms (lower graphs) of the vowel (**A**) and sine tone stimulus (**B**). In this spectrogram, the acoustic energy in a certain frequency band and at a certain time point is proportional to the darkness of the grey shading. (**C, D**) Mean ± SD amplitudes of the four consecutive N1m peaks for the vowel (**C**) and the tone (**D**) conditions are presented (n = 15). The N1m peak amplitudes in the grand average of vowel and tone stimulation are displayed in Figure 2A.

Analysis of variance (ANOVA) did not reveal significant differences between N1m baseline-to-peak amplitudes after stimulation with the vowel /a/ and N1m amplitudes after stimulation with a matched sine tone (Figure 1C and 1D, Tukey's multiple comparison test, *p *> 0.05 for the comparisons N1m_1_(vowel) vs. N1m_1_(tone), N1m_2_(vowel) vs. N1m_2_(tone), N1m_3_(vowel) vs. N1m_3_(tone) and N1m_4_(vowel) vs. N1m_4_(tone)). Thus, a grand average of all responses to vowels and tones was calculated for each individual subject. The combined analysis of both conditions yielded a better signal-to-noise ratio, resulting in a more robust source localisation and a more accurate calculation of the amplitude over time. All further analyses were performed with the grand average across individuals and conditions.

**Figure 2:**
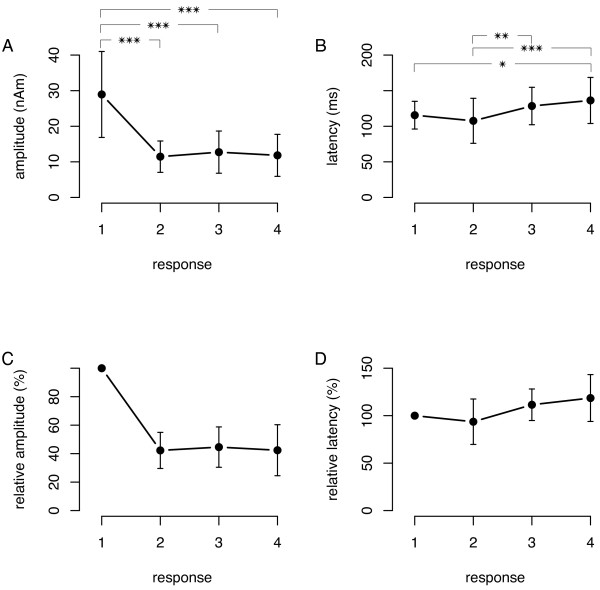
**N1m amplitudes and latencies**. (**A, B**) Mean amplitudes ± SD (**A**) and latencies (**B**) of the four N1m peaks (grand average of vowel and tone stimulation). * *p *< 0.05, * *p *< 0.01, *** *p *< 0.001. (**C, D**) Mean ± SD relative amplitudes (**C**) and relative latencies (**D**) of the four N1m peaks. Amplitudes and latencies are relative to the first N1m peak.

A repeated-measures ANOVA showed that the N1m amplitude decreased with the number of responses (Figure 2A, stimulation with four successive stimuli in a train. *F *= 40.14, *p *< 0.0001). *Post hoc *tests using Tukey's multiple comparison test indicated that the amplitudes of the second, third and fourth N1m were significantly smaller than the amplitude of the first N1m (*p *< 0.001 for each comparison). The relative amplitudes of the second, third, and fourth N1m waves (each normalised to the peak amplitude of the first N1m) are depicted in Figure 2C.

The N1m peak latency significantly increased with the number of responses (Figure 2B, Friedman test, a non-parametric repeated measures ANOVA, *p *< 0.0001). *Post hoc *tests using Dunn's multiple comparison test demonstrated that the latency of the fourth N1m was significantly longer than the latency of the first (*p *< 0.05) and the second N1m (*p *< 0.001). The relative latencies of the second, third, and fourth N1m waves are shown in Figure 2D.

### Magnetic resonance spectroscopy

The concentrations of N-acetylaspartate (NAA) (median 12 institutional units (IU), range 6–20 IU), choline-containing compounds (Cho) (median 1.4 IU, range 0.9–3.0 IU) and the glutamine/glutamate pool (Glx) (median 22, range 4–40 IU) demonstrated considerable intersubject variability.

### Associations between magnetic resonance spectroscopy and magnetoencephalography

**Figure 3:**
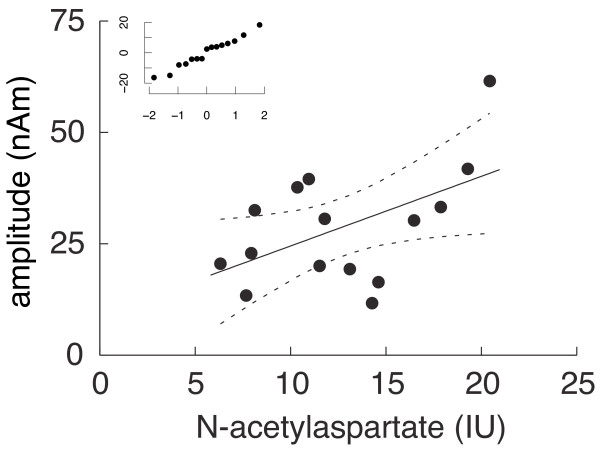
**N1m amplitude as a function of N-acetylaspartate**. Linear regression plot of the amplitude of the first N1m vs. N-acetylaspartate (NAA) (*R*^2 ^= 0.369, *F*(1,13), *p *= 0.016). The 95% confidence intervals are represented by dashed lines. The insert shows the normal probability plot of the residuals, indicating that the errors are normally distributed. IU, institutional unit.

**Figure 4:**
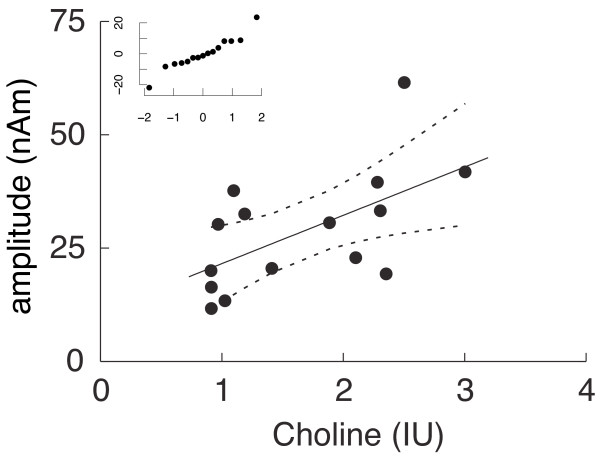
**N1m amplitude as a function of choline**. Linear regression plot of the amplitude of the first N1m vs. choline-containing compounds (Cho) (*R*^2 ^= 0.287, *F*(1,13), *p *= 0.040). The 95% confidence intervals are represented by dashed lines. The insert shows the normal probability plot of the residuals, indicating that the errors are normally distributed. IU, institutional unit.

**Figure 5:**
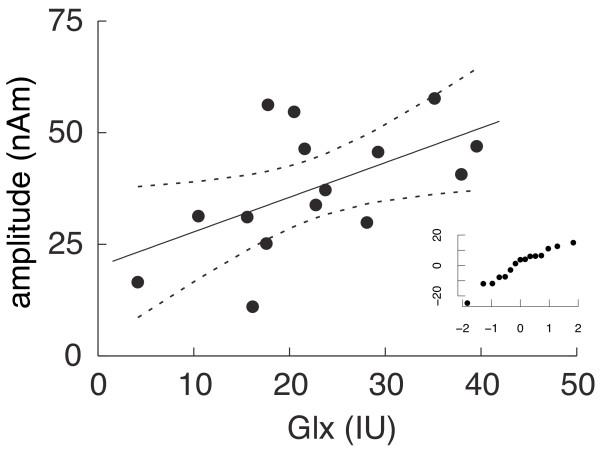
**Relative N1m amplitude as a function of glutamine**. Linear regression plot of the relative amplitude of the second N1m peak vs. the glutamine/glutamate pool (Glx) (*R*^2 ^= 0.26, *F*(1,13), *p *= 0.054). The 95% confidence intervals are represented by dashed lines. The insert shows the normal probability plot of the residuals, indicating that the errors are normally distributed. IU, institutional unit.

The amplitude of the first N1m peak was significantly associated with the concentration of NAA (Figure 3, linear regression, *R*^2 ^= 0.369, *F*(1,13), *p *= 0.016) and Cho (Figure 4, *R*^2 ^= 0.287, *F*(1,13), *p *= 0.040). No significant association was found between Glx and the amplitude of the first N1m. Linear regression analysis revealed no significant associations between the relative amplitude of the second N1m response and NAA, Cho or Glx. However, there was a trend for higher Glx concentrations in individuals with higher relative N1m amplitude (*R*^2 ^= 0.26, *F*(1,13), *p *= 0.054) (Figure 5).

As this study included participants between 18 and 68 years, we addressed the question of whether age might influence the measured variables and the calculated statistical associations. No significant associations were found between age and the tested variables (NAA, Glx, Cho, first N1m amplitude, relative second N1m amplitude; data not shown). In addition, we reanalysed the data after the exclusion of the two oldest subjects (60 and 68 years old). For this subgroup (n = 13, mean age = 27 years), the amplitude of the first N1m peak was associated with the concentration of NAA (*R*^2 ^= 0.324, *F*(1,11), *p *= 0.042) and with the concentration of Cho (*R*^2 ^= 0.437, *F*(1,11), *p *= 0.014). However, no significant association was found between the relative amplitude of the second N1m peak and the concentration of Glx (*R*^2 ^= 0.259, *F*(1,11), *p *= 0.076).

## Discussion

Magnetoencephalographic and MR spectroscopic investigations of 15 adults revealed: (i) a significant association between the amplitudes of individual N1m responses and the concentrations of NAA and Cho and (ii) a trend for higher Glx concentrations in individuals with higher relative N1m amplitude. The present study is, to our knowledge, the first to combine magnetoencephalographic assessment of cortical auditory processing and MR spectroscopic estimation of neurochemicals in the auditory cortex. In an earlier study from our group, auditory MEG and MRS were used independently to investigate electrophysiological and neurochemical changes in patients with depression [18]. In the present investigation, in contrast, we examined the interactions between electrophysiological and neurochemical variables. Our data suggest that the interindividual differences of cortical auditory processing are generated, at least in part, by the variability of neurochemicals within the auditory cortex.

### Auditory evoked magnetic fields

In the present study, no significant differences between the vowel and the sine-tone condition were found regarding the absolute or relative amplitudes of the N1m peak. This result corroborates a previous report in which no significant differences between the amplitudes of the N1m elicited by a sine tone or the vowel /a/ were seen in a passive listening condition (watching a silent movie, as in the present study) [19]. In contrast, vowels evoked a significantly stronger N1m response than tones in a study involving a stimulus detection task [20].

For the present study, we investigated associations between N1m amplitudes, but not N1m latencies, and the neurochemistry of the auditory cortex. We did not assess the influence of MRS variables on N1m latency because this latency depends, in part, on the subcortical auditory pathways, which could not be investigated with ^1^H-MRS, used here.

### Variability of auditory evoked responses

The macroscopic and microscopic variability of primary and secondary auditory cortices has been carefully investigated [21,22]. In particular, the anatomy of the major generator site of the human N1m response, the planum temporale [23], is characterised by extensive interindividual variability [22]. On the individual level, our AEF recordings displayed a pronounced interindividual variability of the peak amplitude and decrement (Figure 2A and 2C). This variability was expected in the light of previous experiments on auditory evoked potentials [24,25].

### Associations between N1m amplitude and neurochemicals

The amino-acid NAA is found almost exclusively in neurons and axons, and can be used as an indirect measure of neuronal integrity and density. Loss of NAA has been reported in a variety of disorders, e.g., in individuals with epilepsy [26], Alzheimer's disease [27] or cognitive impairment [28]. Cho, the choline-containing compounds measurable with ^1^H-MRS, are dominated by phosphocholine, glycerophosphocholine, and free choline [29]. As precursors or degradation products of membrane phospholipids make a major contribution to the Cho signal, Cho is regarded as a membrane marker [30]. In addition, choline is the rate-limiting substrate for the synthesis of the neurotransmitter acetylcholine via the enzyme choline acetyltransferase.

The magnetic dipole moment detected by MEG represents the postsynaptic currents of approximately 1 million synapses activated synchronously. Differences in brain magnetic fields are thus assumed to reflect differences in the number of activated synapses and/or in the coherence of activated neurons [31]. Our data suggest that the number and integrity of neurons (NAA) and the density and the functional integrity of cell membranes (Cho) are associated with the amplitude of auditory evoked responses.

The metabolic variables reported here, however, explain only in part the interindividual differences in cortical auditory processing. The association between NAA and the N1m amplitude accounts for 37% of the measured variance, while the association between Cho and the N1m amplitude accounts for 29% of the variance.

### Associations between N1m decrement and neurochemicals

This study revealed no significant associations between the concentrations of NAA, Cho or Glx and the reduction of the N1m amplitude after a preceding stimulus (the N1m decrement). However, there was a trend for higher Glx concentrations in individuals with higher relative N1m amplitude (i.e., smaller decrement) (Figure 5, *R*^2 ^= 0.26, *F*(1,13), *p *= 0.054).

Previous work on cortical processing of rapidly recurring stimuli indicated that inhibitory interneuronal networks modulate the amplitude and the decrement of cortical evoked responses [3,32]. Several classes of interneurons with distinct functional properties regulate the inhibitory activity in the neocortex [33]. The activity of interneurons appears to be mainly regulated by the major inhibitory neurotransmitter gamma-aminobutyric acid (GABA) [34] and the major excitatory neurotransmitter, glutamate [35].

The concentration of GABA could not be measured by the MRS technique used here. The spectroscopic measurement of GABA *in vivo *is difficult, due to the overlapping peaks of NAA, Glx, and creatine. Although MR protocols have been developed for the assessment of GABA, these techniques require a measurement volume of more than 35 cm^3 ^(compared with 3.375 cm^3 ^here), much larger than the auditory cortex [25].

Glx represents the concentrations of the amino-acid glutamate and its precursor glutamine. In the peripheral and central auditory system, glutamate receptors of the alpha-amino-3-hydroxy-5-methyl-4-isoxazolepropionic acid (AMPA) type play an important role in the rapid synaptic transmission [36]. Moreover, AMPA receptors regulate the processing of rapidly successive stimuli in the peripheral auditory system [37].

### Methodological considerations

The aim of the present study was to correlate electrophysiological parameters of auditory processing with the neurochemistry of the auditory cortex. This study relies on the following assumptions.

#### Reliability of MEG and MRS results

As the MEG and MRS measurements could not be performed simultaneously, the present study relies on the reproducibility of the measured electrophysiologic and neurochemical variables over time. In several investigations using EEG and MEG, a high reproducibility of the N1/N1m amplitude has been found [38,39]. Using the experimental paradigm of this study, replication measurements with two participants were performed. The amplitude of the first N1m response and the response decrement from the first to the second N1m were highly consistent within 3 months. This result corroborates a study by Lütkenhöner et al, who used the same MEG system as used for the present study [14]. Similarly, the intraindividual reproducibility of NAA, Cho and Glx concentrations was demonstrated by our group [40] and by others [41,42].

#### Stability of MRS results under acoustic stimulation

MRS examinations, similar to conventional MRI scans, are associated with intense scanner noise caused by rapidly switching the gradient coils on and off. During the ^1^H-MRS scans here, short (100 ms) periods of scanner noise, repeated every 1.5 seconds, were produced. As the acoustic stimulus during the MEG recording was (necessarily) different, our study relies on the stability of neurochemicals under different kinds of acoustic stimulation. In a functional MRS study comparing scanner noise alone or in combination with siren noise or music, the concentration of lactate changed in relation to acoustic stimulation, while the other spectroscopic parameters remained stable [43]. Similarly, visual stimulation [44] or a silent word-generation task increased brain lactate concentration, but did not change the levels of the other metabolites as measurable by ^1^H-MRS. Thus, it appears unlikely that the noise-induced activation of the auditory cortex during MRS significantly changed the metabolites measured here.

### Directions for future research

Since the advent of non-invasive techniques to investigate human brain function, such as positron emission tomography, functional magnetic resonance imaging, MRS and MEG, numerous studies have been performed using either modality. Recently, an increasing number of researchers have combined neuroimaging modalities in an attempt to overcome the limitations of each of these methods [45,46].

The present study correlated the electrophysiology of auditory processing as recorded by MEG with the neurochemistry of the auditory cortex as assessed by MRS. Our study, however, has several limitations. First, the small number of participants limited the power of the statistical analyses. In addition, we acquired data from only the left hemisphere. The 37-channel MEG system used here could be positioned close to the superior temporal plane, resulting in a good signal-to-noise ratio, but could only record the neural activity of one hemisphere at a time. The processing of acoustic stimuli can differ between hemispheres, especially when speech stimuli are used [47]. Similarly, the concentrations of neurochemicals, as assessed by MRS, may display interhemispheric differences [48]. Therefore, we cannot rule out that MEG-MRS associations for the right auditory cortex differ from those reported here for the left auditory cortex. For future studies, a larger sample size and data collection from both hemispheres (using a whole-head MEG system) are desirable. Moreover, we performed MEG recordings while participants were watching a silent movie (i.e., in a passive listening condition). The associations between MEG and MRS parameters reported here might change when MEG is recorded under top-down attentional modulation, as attention is known to influence auditory processing [49].

In addition to the concentration of NAA and Cho, other factors have been shown to influence auditory cortical processing, such as serotonergic neurotransmission (see Background) [7]. In a study combining electrophysiology and genetics, the amplitude increase of the N1/P2 component in response to increasing stimulus intensities (termed the loudness dependence) was significantly different between individuals with different variants of the serotonin transporter gene. We expect that genetic studies will considerably contribute to future research on individual auditory processing.

## Conclusion

The combined use of MEG and MRS indicated that the processing of rapid auditory stimuli seems to depend, among other factors, on the functional integrity of neurons and cell membranes in the auditory cortex, as reflected by the concentrations of NAA and Cho. This approach provides evidence that individual differences in the neurochemical composition of the cortex account, in part, for individual electrophysiological differences in auditory processing.

## Methods

### Participants

Fifteen healthy volunteers (six women, nine men) with a median age of 24 years (range: 18–68 years) participated in this study. Two subjects were 60 years or older, the remaining 13 were 54 years or younger. Fourteen participants were right-handed, one was left-handed [50]. All participants had a normal audiological status and were without a history of neurological or otological disorders. All individuals gave their informed consent to participate in the study. This project was reviewed and approved by the Research Ethics Board, Medical Faculty, University of Münster. The participants of this study had served as healthy controls in a previous study on metabolic and electrophysiological changes in patients with depression [18]. MEG recordings of subgroups of the participants in this study were also published separately [51,52].

### Magnetic resonance spectroscopy

**Figure 6:**
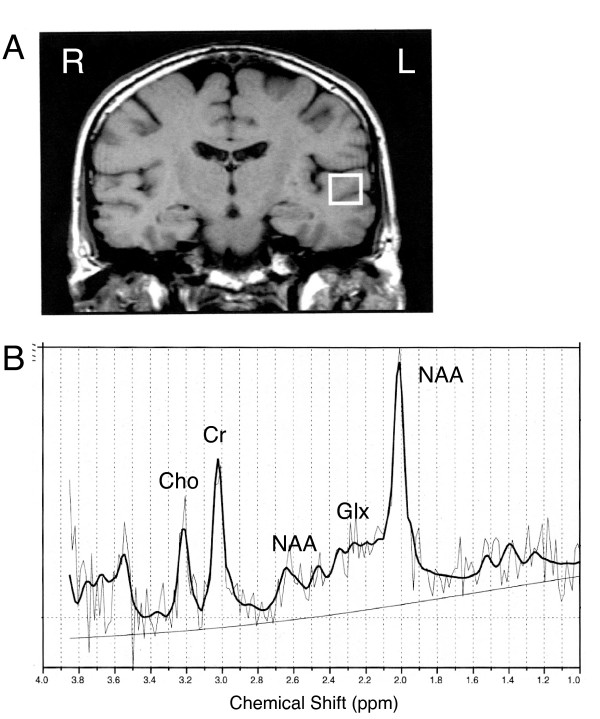
**MR spectroscopy of the auditory cortex**. (**A**) Anatomical transverse MR image of the left auditory cortex with the superimposed borders of the MRS voxel (1.5 × 1.5 × 1.5 cm^3^). The image shows the anterior borders of the voxel, which extends 1.5 cm in the posterior direction. (**B**) ^1^H-MRS spectrum from the auditory cortex of a single subject. NAA denotes N-acetylaspartate; Glx, glutamate/glutamine; Cr, creatine/phosphocreatine; Cho, choline-containing compounds. The original and the fitted spectrum (using the linear combination model, black line) are displayed.

To investigate individual brain anatomy and to exclude possible brain lesions, structural MR images were obtained from every subject. T1-weighted, three-dimensional, spoiled gradient echo MRI of the whole brain and T2- and proton density-weighted fast spin echo images in axial and coronal orientation were performed on a 1.5-Tesla scanner (Magnetom SP, Siemens, Erlangen, Germany). To assess the concentrations of brain metabolites, single voxel stimulated echo acquisition mode (STEAM) spectroscopy was used (echo time, TE = 20 ms; repetition time, TR = 2.5 s; number of scans = 128) [53]. Using the individual structural MRI, the MRS voxel was centred at the left transverse gyrus of Heschl (Brodmann area 41) on the dorsal surface of the superior temporal plane containing the primary and secondary human auditory cortex. The MRS voxel volume was 1.5 × 1.5 × 1.5 cm^3 ^(Figure 6).

### MRS postprocessing

The postprocessing of MRS spectra was performed as described previously [40,54,55]. Concentrations of N-acetylaspartate (NAA), choline-containing compounds (Cho) and the glutamine/glutamate pool (Glx) were quantified using a time domain fitting program, based on the linear combination (LC) model [56]. Owing to the overlapping resonances of glutamate, glutamine and GABA at a field strength of 1.5 T, a combined fitting of the glutamine/glutamate pool was performed. Metabolite concentrations, acquired by the LC model and scaled to water, were corrected for coil loading [57] and partial volume effects [58]. To account for variations in voxel composition (cerebrospinal fluid, grey matter and white matter), brain segmentation was performed with a semiautomatic interactive algorithm (M. Fiebich, University of Applied Sciences, Giessen, Germany) [54]. Metabolites were then normalised to the grey-matter fraction in the voxels, and expressed in institutional units (IU). For robustness of results, only data with a fitting error <20% of the standard deviation was included in the final analysis.

### Magnetoencephalographic measurement

#### Auditory stimulation

**Figure 7:**
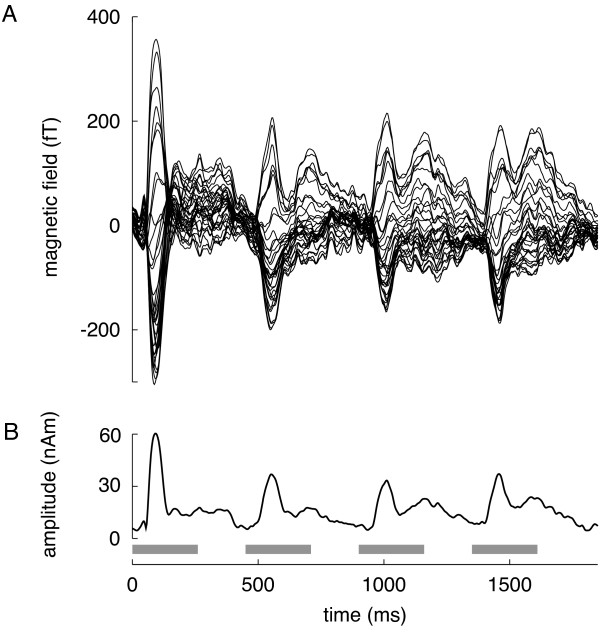
**Auditory evoked magnetic fields**. (**A**) Magnetic waveforms of an individual subject, superimposition of 37 MEG channels, covering the left hemisphere. (**B**) Amplitude of the four N1m peaks in the same subject. The grey bars indicate the onset and duration of the acoustic stimulation.

Short sequences of rapidly recurring speech and non-speech sounds served as stimuli. The German vowel /a/ (duration 260 ms, fundamental frequency f0 234 Hz), spoken by a female speech-language pathologist, and a matched sine tone were chosen (frequency 234 Hz, duration 260 ms). The envelope of the sine tone was adjusted to the envelope of the vowel /a/. Sequences of four successive stimuli (either four vowels or four tones) were presented with an onset-to-onset interstimulus interval of 450 ms (Figure 7). As electromagnetic activation of the auditory cortex lasts for approximately 400 ms after the onset of a single transient stimulus, an interstimulus interval of 450 ms (or 2.2 stimuli/second) was chosen to avoid overlap between successive brain responses. The onset-to-onset interval between sequences was 4.5 seconds (randomised between 4 and 5 seconds). This interval is long enough to allow a substantial recovery of the N1m component before the onset of the following train [59]. Participants listened passively to 160 trials of the vowel stimulation and to 160 trials of the tone stimulation in randomised order.

#### MEG data acquisition

AEFs were recorded with a 37-channel axial gradiometer system (Magnes I; BTi, San Diego, USA) in a magnetically shielded room and sampled at rate of 512.4 Hz. The participants were in a right lateral position with the body supported by a vacuum cushion to minimise head and body movements during the measurement. The sensor array was positioned over the auditory cortex as closely as possible to the subject's head. To ensure a stable passive listening condition, subjects watched a self-selected silent video that attracted their attention. Participants were instructed not to move their head, to stay awake, and to keep their eyes open. Immediately before each MEG measurement, the individual hearing thresholds were determined for the vowel and the tone stimuli separately. The stimuli were delivered to a silicon earpiece in the right ear via speakers outside the shielded room and a plastic tube of 6.3 m length. The stimulation system was able to transmit frequencies up to approximately 4500 Hz [60]. All stimuli were presented with an intensity of 60 dB above the individual hearing threshold.

#### MEG data analysis

Artefact-contaminated epochs were excluded if the magnetic field value exceeded the baseline value (calculated in the time window of -250 to 0 ms before stimulus onset) by 2 pT. Between 4% and 13% of the data had to be discarded. After exclusion of artefacts, three averaged datasets were created per subject: (i) vowels alone, (ii) sine tones alone, and (iii) vowels and sine tones combined. All datasets were baseline corrected (-250 to 0 ms before the first stimulus) and filtered with a band-pass filter of 0.01–40 Hz. Figure 7A shows the overlay of the recordings of all 37 channels in an individual participant. To identify the successive N1m peaks, the root mean square of amplitudes was calculated for a time window of 0 to 2000 ms, based on the amplitudes in each of the 37 MEG channels. To obtain a dipole that was representative of the N1m, we first calculated a single equivalent current dipole for each sampling point and then averaged the dipole parameters for a 30-ms time window around the activity peak (response to the first stimulus of series, as determined by root mean square of amplitude curves). This N1m dipole was finally used to calculate the dipole moment over the entire epoch. Figure 7B displays the dipole moment as a function of time in an individual participant. For this calculation, the location and the direction of the N1m dipole were assumed to be constant (fixed-dipole approach). The strongest deflections in the latency range of the N1m were identified as N1m responses. Based on the dipole moment over time, the peak amplitudes and peak latencies of the N1m responses were determined. In most participants, a baseline shift was detected from the beginning of the first response to the fourth response. To ensure accurate quantification of amplitudes, dipole moments were measured relative to a baseline that was defined as the mean value before the onset of each stimulus (-50 to 0 ms prestimulus). The amplitude ratio of the second and first N1m (termed the relative amplitude of the second N1m) was then calculated.

### Assessment of test-retest reliability

To test for reliability of MRS data, we measured six healthy individuals twice, at a mean ± SD of 5.2 ± 0.8 months apart, in a separate study [40]. Repeated-measures ANOVA yielded no significant difference between Glx concentrations of the left dorsolateral prefrontal cortex in the first and second measurement (*F *= 0.39, *p *= 0.56). Glx concentrations are usually responsible for the largest variance in MRS data (B. Pfleiderer, unpublished observation). The coefficient of variation for Glx in our previous study [40] was 12%. These values are similar to the coefficients of variation, ranging from 13% to 15%, which were previously reported for ^1^H chemical shift imaging experiments [61]. These results indicate that the metabolite concentrations measured here are reasonably stable over time.

**Figure 8:**
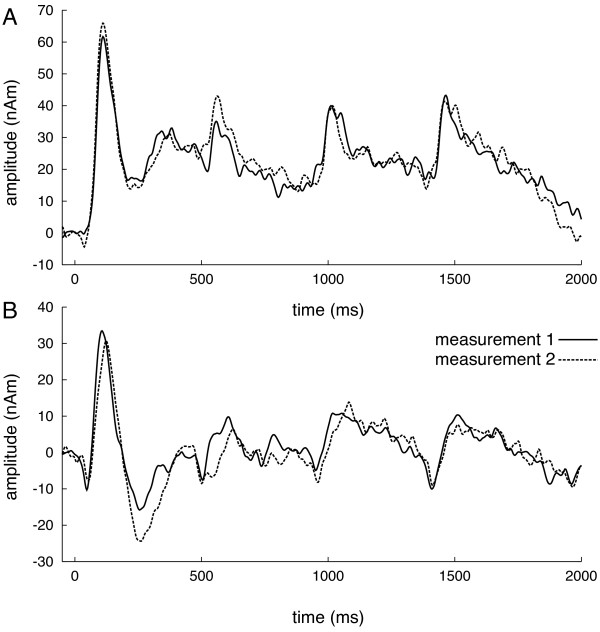
**Reliability of auditory evoked field recordings**. (**A, B**) Amplitudes of two AEF measurements, 4 months apart, in two different subjects.

To assess the reliability of AEF recordings, measurements were repeated in two participants after 4 months (Figure 8). The overall correlation between amplitudes over time in the first and the second recording were high (participant 1: r = 0.93, participant 2: r = 0.87). Most important for this study, the first N1m responses were almost identical. The reliability of later responses, in particular the second N1m, was smaller. These measurements corroborate an earlier study on the reliability of N1m recordings [14].

### Statistical analysis

To test if the collected data were normally distributed, the Shapiro-Wilk normality test was performed [62]. This test is widely used to test for normality in smaller samples because of its high statistical power compared with alternative tests [63]. The assumption of a normal distribution was rejected if *p *< 0.01. According to this test, all electrophysiological and neurochemical variables were normally distributed, except the latencies of the four N1m responses. To compare the absolute and relative amplitudes of the four successive N1m waves, a repeated-measures ANOVA with Tukey's multiple-comparison *post hoc *test was calculated (Figure 2A). A nonparametric repeated-measures ANOVA (Friedman test with Dunn's multiple comparison *post hoc *test) was performed to compare the absolute and relative latencies of the four N1m waves (Figure 2B). To assess the relationship between absolute and relative N1m amplitudes and neurochemical parameters, a linear regression analysis was performed (Figures 3, 4, 5). Linear-regression models assume that the unpredicted variation (the error term ε) is normally distributed. To test for the normality of errors, a normal-probability plot of the residuals was performed (insets in Figures 3, 4, 5). If the error distribution is normal, the points on the normal-probability plot should fall close to a diagonal line. The linear-regression model was used to fit a regression line through the data and to calculate the 95% confidence intervals (Figures 3, 4, 5). Statistical analyses were performed with the statistical package R for Mac OS X [64].

## Authors' contributions

PS and NM contributed equally to this work. PS, NM and BP designed the study and drafted the manuscript. PS and MT recorded and analysed MEG data. BP and MT performed and analysed MRS scans. All authors read and approved the final manuscript.

## References

[B1] Fruhstorfer H, Soveri P, Järvilehto T (1970). Short-term habituation of the auditory evoked response in man. Electroencephalogr Clin Neurophysiol.

[B2] Hari R, Kaila K, Katila T, Tuomisto T, Varpula T (1982). Interstimulus interval dependence of the auditory vertex response and its magnetic counterpart: implications for their neural generation. Electroencephalogr Clin Neurophysiol.

[B3] Okamoto H, Kakigi R, Gunji A, Kubo T, Pantev C (2005). The dependence of the auditory evoked N1m decrement on the bandwidth of preceding notch-filtered noise. Eur J Neurosci.

[B4] Michael N, Ostermann J, Sörös P, Schwindt W, Pfleiderer B (2004). Altered habituation in the auditory cortex in a subgroup of depressed patients by functional magnetic resonance imaging. Neuropsychobiology.

[B5] Pfleiderer B, Ostermann J, Michael N, Heindel W (2002). Visualization of auditory habituation by fMRI. Neuroimage.

[B6] Grunwald T, Boutros N, Pezer N, von Oertzen J, Fernández G, Schaller C, Elger C (2003). Neuronal substrates of sensory gating within the human brain. Biol Psychiatry.

[B7] Kähkönen S (2006). Magnetoencephalography (MEG): a non-invasive tool for studying cortical effects in psychopharmacology. Int J Neuropsychopharmacol.

[B8] Kähkönen S, Ahveninen J, Jääskeläinen I, Kaakkola S, Näätänen R, Huttunen J, Pekkonen E (2001). Effects of haloperidol on selective attention: a combined whole-head MEG and high-resolution EEG study. Neuropsychopharmacology.

[B9] Pekkonen E, Hirvonen J, Jääskeläinen I, Kaakkola S, Huttunen J (2001). Auditory sensory memory and the cholinergic system: implications for Alzheimer's disease. Neuroimage.

[B10] Pekkonen E, Jääskeläinen I, Kaakkola S, Ahveninen J (2005). Cholinergic modulation of preattentive auditory processing in aging. Neuroimage.

[B11] Kähkönen S, Marttinen Rossi E, Yamashita H (2005). Alcohol impairs auditory processing of frequency changes and novel sounds: a combined MEG and EEG study. Psychopharmacology (Berl).

[B12] Umbricht D, Schmid L, Koller R, Vollenweider F, Hell D, Javitt D (2000). Ketamine-induced deficits in auditory and visual context-dependent processing in healthy volunteers: implications for models of cognitive deficits in schizophrenia. Arch Gen Psychiatry.

[B13] Manjarrez G, Hernandez E, Robles A, Hernandez J (2005). N1/P2 component of auditory evoked potential reflect changes of the brain serotonin biosynthesis in rats. Nutr Neurosci.

[B14] Lütkenhöner B (1998). Dipole source localization by means of maximum likelihood estimation. II. Experimental evaluation. Electroencephalogr Clin Neurophysiol.

[B15] Hari R, Levänen S, Raij T (2000). Timing of human cortical functions during cognition: role of MEG. Trends Cogn Sci.

[B16] Godey B, Schwartz D, deGraaf J, Chauvel P, Liégeois-Chauvel C (2001). Neuromagnetic source localization of auditory evoked fields and intracerebral evoked potentials: a comparison of data in the same patients. Clin Neurophysiol.

[B17] Rothman D, Behar K, Hyder F, Shulman R (2003). In vivo NMR studies of the glutamate neurotransmitter flux and neuroenergetics: implications for brain function. Annu Rev Physiol.

[B18] Tollkötter M, Pfleiderer B, Sörös P, Michael N (2006). Effects of antidepressive therapy on auditory processing in severely depressed patients: A combined MRS and MEG study. J Psychiatr Res.

[B19] Tiitinen H, Sivonen P, Alku P, Virtanen J, Näätänen R (1999). Electromagnetic recordings reveal latency differences in speech and tone processing in humans. Brain Res Cogn Brain Res.

[B20] Gootjes L, Raij T, Salmelin R, Hari R (1999). Left-hemisphere dominance for processing of vowels: a whole-scalp neuromagnetic study. Neuroreport.

[B21] Rademacher J, Morosan P, Schormann T, Schleicher A, Werner C, Freund H, Zilles K (2001). Probabilistic mapping and volume measurement of human primary auditory cortex. Neuroimage.

[B22] Westbury C, Zatorre R, Evans A (1999). Quantifying variability in the planum temporale: a probability map. Cereb Cortex.

[B23] Lütkenhöner B, Steinsträter O (1998). High-precision neuromagnetic study of the functional organization of the human auditory cortex. Audiol Neurootol.

[B24] Lauter J, Karzon R (1990). Individual differences in auditory electric responses: comparisons of between-subject and within-subject variability. V. Amplitude-variability comparisons in early, middle, and late responses. Scand Audiol.

[B25] Lütkenhöner B, Krumbholz K, Seither-Preisler A (2003). Studies of tonotopy based on wave N100 of the auditory evoked field are problematic. Neuroimage.

[B26] Mueller S, Suhy J, Laxer K, Flenniken D, Axelrad J, Capizzano A, Weiner M (2002). Reduced extrahippocampal NAA in mesial temporal lobe epilepsy. Epilepsia.

[B27] Schuff N, Capizzano A, Du A, Amend D, O'Neill J, Norman D, Kramer J, Jagust W, Miller B, Wolkowitz O, Yaffe K, Weiner M (2002). Selective reduction of N-acetylaspartate in medial temporal and parietal lobes in AD. Neurology.

[B28] Chao L, Schuff N, Kramer J, Du A, Capizzano A, O'Neill J, Wolkowitz O, Jagust W, Chui H, Miller B, Yaffe K, Weiner M (2005). Reduced medial temporal lobe N-acetylaspartate in cognitively impaired but nondemented patients. Neurology.

[B29] Miller B, Chang L, Booth R, Ernst T, Cornford M, Nikas D, McBride D, Jenden D (1996). In vivo 1H MRS choline: correlation with in vitro chemistry/histology. Life Sci.

[B30] Boulanger Y, Labelle M, Khiat A (2000). Role of phospholipase A(2) on the variations of the choline signal intensity observed by 1H magnetic resonance spectroscopy in brain diseases. Brain Res Brain Res Rev.

[B31] Lü Z, Williamson S (1991). Spatial extent of coherent sensory-evoked cortical activity. Exp Brain Res.

[B32] Miller C, Freedman R (1995). The activity of hippocampal interneurons and pyramidal cells during the response of the hippocampus to repeated auditory stimuli. Neuroscience.

[B33] McLean M, Busza A, Wald L, Simister R, Barker G, Williams S (2002). In vivo GABA+ measurement at 1.5T using a PRESS-localized double quantum filter. Magn Reson Med.

[B34] Blatow M, Caputi A, Monyer H (2005). Molecular diversity of neocortical GABAergic interneurones. J Physiol.

[B35] Virtanen J, Ahveninen J, Ilmoniemi R, Näätänen R, Pekkonen E (1998). Replicability of MEG and EEG measures of the auditory N1/N1m-response. Electroencephalogr Clin Neurophysiol.

[B36] Parks T (2000). The AMPA receptors of auditory neurons. Hear Res.

[B37] Duan M, Canlon B (2001). Short-term adaptation in the peripheral auditory system is related to the AMPA receptor. Acta Otolaryngol.

[B38] Rotondo E, Bruschetta G, Saccà A, Bramanti P, Di Pasquale M (2003). Straightforward relative quantitation and age-related human standards of N-acetylaspartate at the centrum semiovale level by CSI (1)H-MRS. Magn Reson Imaging.

[B39] Tedeschi G, Bertolino A, Campbell G, Barnett A, Duyn J, Jacob P, Moonen C, Alger J, Di Chiro G (1996). Reproducibility of proton MR spectroscopic imaging findings. AJNR Am J Neuroradiol.

[B40] Michael N, Gösling M, Reutemann M, Kersting A, Heindel W, Arolt V, Pfleiderer B (2003). Metabolic changes after repetitive transcranial magnetic stimulation (rTMS) of the left prefrontal cortex: a sham-controlled proton magnetic resonance spectroscopy (1H MRS) study of healthy brain. Eur J Neurosci.

[B41] Prichard J, Rothman D, Novotny E, Petroff O, Kuwabara T, Avison M, Howseman A, Hanstock C, Shulman R (1991). Lactate rise detected by 1H NMR in human visual cortex during physiologic stimulation. Proc Natl Acad Sci USA.

[B42] Richards T, Gates G, Gardner J, Merrill T, Hayes C, Panagiotides H, Serafini S, Rubel E (1997). Functional MR spectroscopy of the auditory cortex in healthy subjects and patients with sudden hearing loss. AJNR Am J Neuroradiol.

[B43] Urrila A, Hakkarainen A, Heikkinen S, Vuori K, Stenberg D, Häkkinen A, Lundbom N, Porkka-Heiskanen T (2003). Metabolic imaging of human cognition: an fMRI/1H-MRS study of brain lactate response to silent word generation. J Cereb Blood Flow Metab.

[B44] Zaehle T, Wüstenberg T, Meyer M, Jäncke L (2004). Evidence for rapid auditory perception as the foundation of speech processing: a sparse temporal sampling fMRI study. Eur J Neurosci.

[B45] Kamada K, Houkin K, Takeuchi F, Ishii N, Ikeda J, Sawamura Y, Kuriki S, Kawaguchi H, Iwasaki Y (2003). Visualization of the eloquent motor system by integration of MEG, functional, and anisotropic diffusion-weighted MRI in functional neuronavigation. Surg Neurol.

[B46] Mathiak K, Fallgatter A (2005). Combining magnetoencephalography and functional magnetic resonance imaging. Int Rev Neurobiol.

[B47] Tiitinen H, Mäkelä A, Mäkinen V, May P, Alku P (2005). Disentangling the effects of phonation and articulation: hemispheric asymmetries in the auditory N1m response of the human brain. BMC Neurosci.

[B48] Jayasundar R, Raghunathan P (1997). Evidence for left-right asymmetries in the proton MRS of brain in normal volunteers. Magn Reson Imaging.

[B49] Obleser J, Elbert T, Eulitz C (2004). Attentional influences on functional mapping of speech sounds in human auditory cortex. BMC Neurosci.

[B50] Oldfield R (1971). The assessment and analysis of handedness: the Edinburgh inventory. Neuropsychologia.

[B51] Teismann I, Sörös P, Manemann E, Ross B, Pantev C, Knecht S (2004). Responsiveness to repeated speech stimuli persists in left but not right auditory cortex. Neuroreport.

[B52] Sörös P, Dziewas R, Manemann E, Teismann IK, Lütkenhöner B (2006). No indication of brain reorganization after unilateral ischemic lesions of the auditory cortex. Neurology.

[B53] Haase A, Frahm J, Matthaei D, Hänicke W, Bomsdorf H, Kunz D, Tischler R (1986). MR imaging using stimulated echoes (STEAM). Radiology.

[B54] Pfleiderer B, Michael N, Gösling M, Erfurth A, Wolgast M, Heindel W (2003). Altered glutamate/glutamine levels within the left dorsolateral prefrontal cortex in aute mania – impact of tissue segmentation and related correlations. Proeceedings of the International Society for Magnetic Resonance in Medicine, 11th Scientific Meeting, Toronto, Canada.

[B55] Pfleiderer B, Ohrmann P, Suslow T, Wolgast M, Gerlach A, Heindel W, Michael N (2004). N-acetylaspartate levels of left frontal cortex are associated with verbal intelligence in women but not in men: a proton magnetic resonance spectroscopy study. Neuroscience.

[B56] Provencher S (2001). Automatic quantitation of localized in vivo 1H spectra with LCModel. NMR Biomed.

[B57] Michaelis T, Merboldt K, Bruhn H, Hänicke W, Frahm J (1993). Absolute concentrations of metabolites in the adult human brain in vivo: quantification of localized proton MR spectra. Radiology.

[B58] Weber-Fahr W, Ende G, Braus D, Bachert P, Soher B, Henn F, Büchel C (2002). A fully automated method for tissue segmentation and CSF-correction of proton MRSI metabolites corroborates abnormal hippocampal NAA in schizophrenia. Neuroimage.

[B59] Woods D, Elmasian R (1986). The habituation of event-related potentials to speech sounds and tones. Electroencephalogr Clin Neurophysiol.

[B60] Lütkenhöner B, Seither-Preisler A, Seither S (2006). Piano tones evoke stronger magnetic fields than pure tones or noise, both in musicians and non-musicians. Neuroimage.

[B61] Chard D, McLean M, Parker G, MacManus D, Miller D (2002). Reproducibility of in vivo metabolite quantification with proton magnetic resonance spectroscopic imaging. J Magn Reson Imaging.

[B62] Shapiro S, Wilk M (1965). An analysis of variance test for normality (complete samples). Biometrika.

[B63] Shapiro S, Wilk M, Chen HJ (1968). A comparative study of various tests of normality. J Am Statistical Assoc.

[B64] R Development Core Team (2005). R: A language and environment for statistical computing.

